# Paraganglioma in the bladder: a case report

**DOI:** 10.1186/s13256-017-1473-2

**Published:** 2017-10-31

**Authors:** Genta Iwamoto, Takashi Kawahara, Mikiko Tanabe, Sahoko Ninomiya, Daiji Takamoto, Taku Mochizuki, Shinnosuke Kuroda, Teppei Takeshima, Koji Izumi, Yusuke Hattori, Jun-ichi Teranishi, Yasushi Yumura, Yasuhide Miyoshi, Hiroji Uemura

**Affiliations:** 1 Departments of Urology and Renal Transplantation, Yokohama City University Medical Center, Yokohama, Japan; 2 Division of Diagnostic Pathology, Yokohama City University Medical Center, Yokohama, Japan

**Keywords:** Paraganglioma, Bladder tumor, Hypertension

## Abstract

**Background:**

Paraganglioma is an extra site of pheochromocytoma. Paraganglioma in the bladder is a very rare disease accounting for 0.06% of all bladder tumors.

**Case presentation:**

A 77-year-old Japanese man was referred to our department for the further examination of a bladder tumor detected on preoperative computed tomography of his gastric cancer. Cystoscopy revealed a submucosal tumor in the upper area of his bladder, so transurethral resection of the bladder tumor was performed. During transurethral resection of the bladder tumor, his blood pressure sharply increased, and a pathological examination showed paraganglioma in his bladder. Postoperative I-123-metaiodobenzylguanidine scintigraphy detected a higher intake of his bladder tumor. Laboratory examinations showed a slightly increased noradrenaline level of 530 pg/ml and reduced platelet count at 167,000/μL. Based on the progression of his gastric cancer, no additional therapy was performed on his bladder tumor. Eight months after surgery, he died from aspiration pneumonitis.

**Conclusions:**

Here we report a rare case of paraganglioma in the bladder. We discuss paraganglioma based on previous studies.

## Background

Paraganglioma is an extra site of pheochromocytoma from the adrenal gland. Paraganglioma is reported to account for 18% of pheochromocytomas, and 10% of cases occur in the bladder. Paraganglioma in the bladder is a very rare disease and accounts for 0.06% of all bladder tumors [1–4].

We performed transurethral resection of bladder tumor (TUR-Bt) on a mass unexpectedly detected on preoperative computed tomography (CT) of a patient’s gastric cancer, and his arterial blood pressure was abnormally elevated during the procedure. Postoperatively, the tumor was diagnosed as paraganglioma. Here we report a rare case of paraganglioma in the bladder.

## Case presentation

**Fig. 1:**
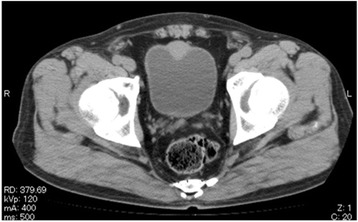
Computed tomography imaging findings of the bladder

**Fig. 2:**
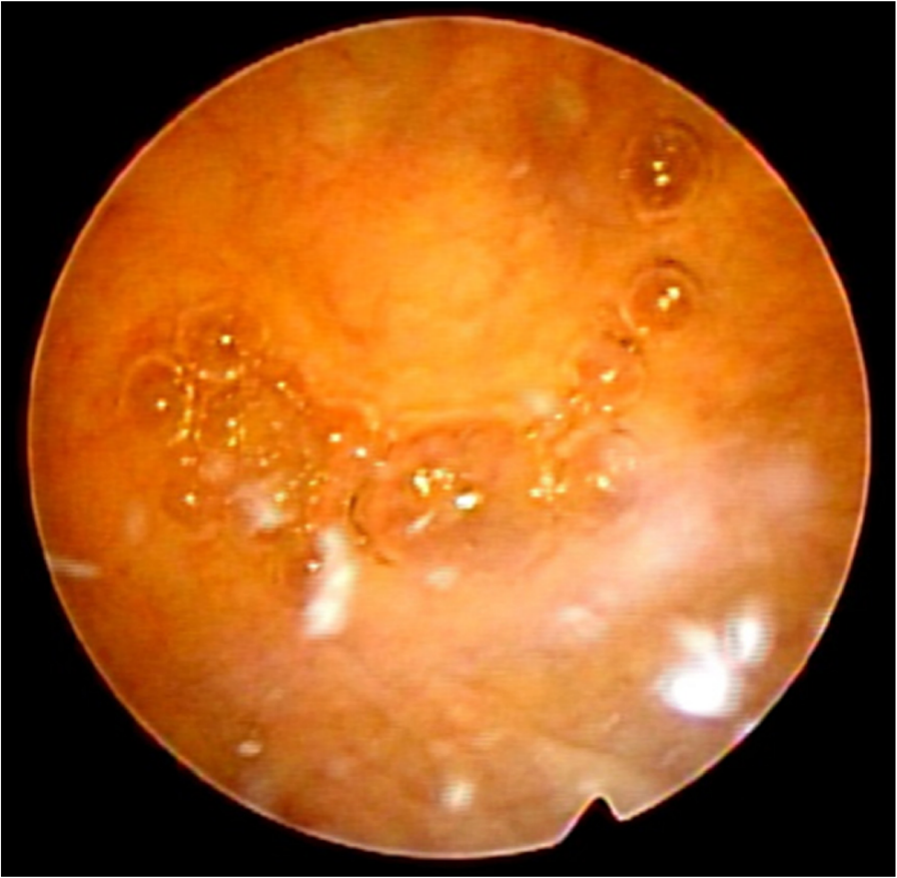
Cystoscopy image. The tumor was covered with urothelial membrane

**Fig. 3:**
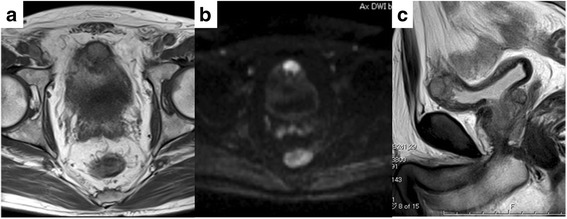
Magnetic resonance imaging in **a** axial T1-weighted imaging, **b** axial diffusion, and **c** sagittal T2-weighted imaging

A 77-year-old Japanese man was referred to our department for treatment of a bladder mass (26 mm in diameter) detected on preoperative CT of his gastric cancer. He had no remarkable medical history except for gastric cancer and hypertension. The stage of gastric cancer was T1b(SM)N0M0. A laboratory examination showed almost normal values except for a low platelet count of 167,000/μL. Urinary cytology showed class II. A CT revealed a 26-mm bladder tumor in the upper region of his bladder (Fig. 1). Cystoscopy showed a non-papillary broad-base tumor surrounded by smooth bladder mucosa (Fig. 2). Magnetic resonance imaging (MRI) suggested muscle invasive bladder cancer with low intensity on T1-weighted and T2-weighted imaging (WI) but high intensity on diffusion-weighted imaging (DWI; Fig. 3).

Based on these findings, TUR-Bt was performed for his bladder tumor. His usual blood pressure was around 120/80 mmHg with olmesartan medoxomil/azelnidipine intake. At the time of starting transurethral resection (TUR), his blood pressure was elevated as 240 mmHg in his systolic blood pressure. In the procedure of TUR, no active bleeding was observed.

**Fig. 4:**
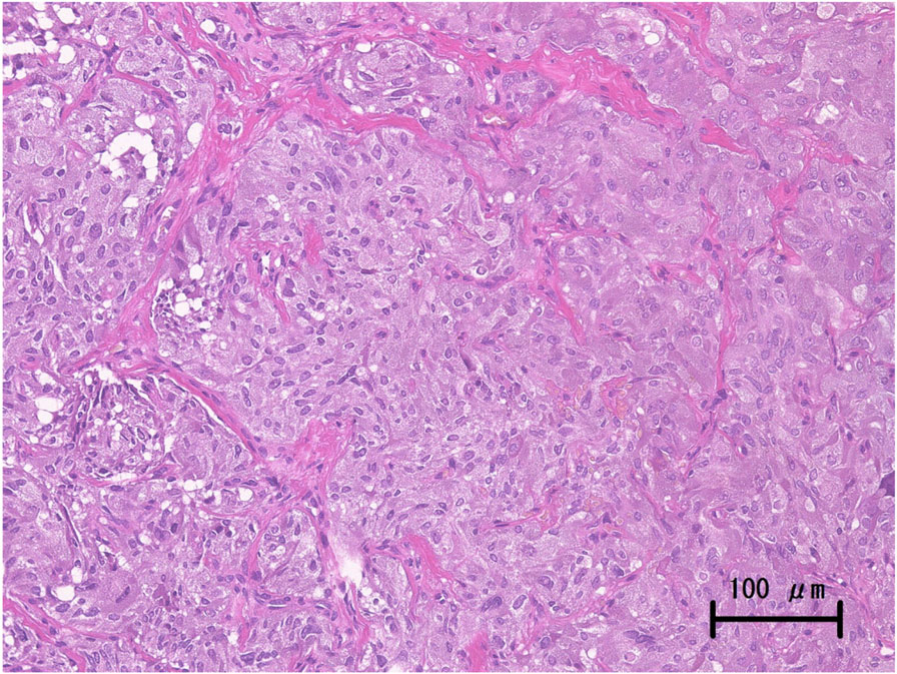
Hematoxylin eosine staining

**Fig. 5:**
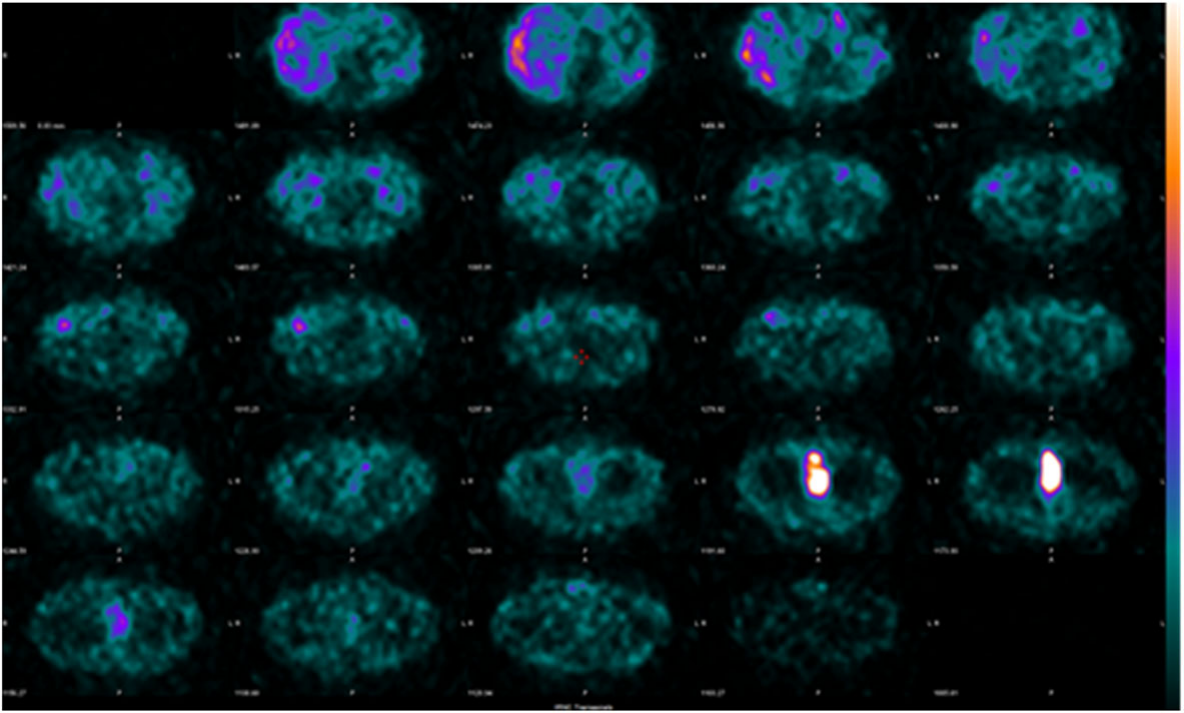
Metaiodobenzylguanidine scintigraphy

Hematoxylin and eosin staining showed (Fig 4) polygonal tumor cells with finely granular cytoplasm were present in the submucosa. Mitotic figure was inconspicuous. An immunohistochemical study revealed negative findings for cytokeratins and positive findings for chromogranin A, synaptophysin, and CD56. Only a few tumor cells showed positive immunolabeling for Ki67. Based on these findings, the pathological examination revealed paraganglioma in his bladder. I-23-metaiodobenzylguanidine (MIBG) scintigraphy showed the residual tumor, but no other uptake was observed (Fig. 5). His serum noradrenaline levels were slightly elevated at 530 pg/mL, but both serum and urine catecholamine showed normal values. Careful observation or partial cystectomy was considered an option for this case because of the lack of muscle invasion by the bladder tumor and pathological results. Although he was scheduled to receive partial cystectomy, he died 8 months after TUR-Bt due to aspiration pneumonitis.

## Discussion

Paraganglioma is a rare neuroendocrine neoplasm which tends to develop in the extra-adrenal glands. Paraganglioma in the bladder is extremely rare and accounts for 0.06% of all bladder tumors [1, 2].

Das *et al*. reported that the symptoms of paraganglioma in the bladder were macroscopic hematuria, hypertension, and hypertension seizure at urination [5]. Yamamoto *et al*. examined 234 Japanese cases and found that 41.3% had macroscopic hematuria, 33.2% hypertension, and 23.0% hypertension seizure at urination. Only 1.4% showed all three symptoms, and 10.6% showed none of these symptoms [6]. The present case showed only hypertension.

MRI and MIBG scintigraphy have been reported useful for a diagnosis. In a previous report, MRI T1-WI showed low intensity, while T2-WI showed high intensity, but not all cases show this pattern [7]. In fact, the present case showed low intensity on both T1-WI and T2-WI.

Regarding laboratory findings, 88% of cases of paraganglioma show elevated levels of urine metanephrine and serum catecholamine [8, 9]. In bladder paraganglioma, more than 60% of cases show elevated levels of urine vanillylmandelic acid or serum catecholamine. Despite these high rates of elevation, a differential diagnosis is sometimes difficult because some cases do not show such elevation [5]. In fact, in the present case, catecholamine was not elevated. As such, the preoperative diagnosis is thought to be difficult in cases of bladder paraganglioma. In a previous study, 28.9% of cases of bladder paraganglioma were diagnosed before surgery, and 61.6% were initially suspected to be bladder tumor or intramucosal bladder tumor [6]. In the present case, the extremely elevated blood pressure during resection and the pathological findings helped us to arrive at the final diagnosis of bladder paraganglioma.

Paraganglioma in the bladder can be benign or malignant, but its histopathological diagnosis is difficult. The diagnosis of benign or malignant status depends on the degree of invasion, lymph node metastasis, and distant metastasis. Our patient had T1b(SM)N0M0 gastric cancer and no previous study showed a correlation between gastric cancer and paraganglioma. Partial cystectomy has been recommended, but TUR-Bt might be an option in cases in which the tumor has not invaded the surrounding fat [10, 11].

## Conclusions

We describe the case of a patient whose blood pressure became extremely elevated during TUR-Bt, and a pathological examination showed paraganglioma of the bladder. Here we report a rare case of paraganglioma in the bladder.
